# An *RGS2* 3′UTR polymorphism is associated with preeclampsia in overweight women

**DOI:** 10.1186/s12863-016-0428-8

**Published:** 2016-08-24

**Authors:** Tiina Karppanen, Tea Kaartokallio, Miira M. Klemetti, Seppo Heinonen, Eero Kajantie, Juha Kere, Katja Kivinen, Anneli Pouta, Anne Cathrine Staff, Hannele Laivuori

**Affiliations:** 1 Medical and Clinical Genetics, University of Helsinki and Helsinki University Hospital, Helsinki, Finland; 2 Obstetrics and Gynecology, University of Helsinki and Helsinki University Hospital, Helsinki, Finland; 3 Department of Obstetrics and Gynecology, South-Karelia Central Hospital, Lappeenranta, Finland; 4 Chronic Disease Prevention Unit, National Institute for Health and Welfare, Helsinki, Finland; 5 Children’s Hospital, Helsinki University Central Hospital and University of Helsinki, Helsinki, Finland; 6 PEDEGO Research Unit, MRC Oulu, Oulu University Hospital and University of Oulu, Oulu, Finland; 7 Department of Biosciences and Nutrition, and Science for Life Laboratory, Karolinska Institutet, Stockholm, Sweden; 8 Folkhälsan Institute of Genetics, Helsinki, Finland; 9 Division of Cardiovascular Medicine, University of Cambridge, Cambridge, UK; 10 Department of Government services, National Institute for Health and Welfare, Helsinki, Finland; 11 Faculty of Medicine, University of Oslo, Oslo, Norway; 12 Department of Obstetrics and Gynecology, Oslo University Hospital, Oslo, Norway; 13 Institute for Molecular Medicine Finland, University of Helsinki, Helsinki, Finland

**Keywords:** Preeclampsia, Pregnancy, Regulator of G-protein signaling 2, Candidate gene study

## Abstract

**Background:**

Preeclampsia is a common and heterogeneous vascular syndrome of pregnancy. Its genetic risk profile is yet unknown and may vary between individuals and populations. The rs4606 3′ UTR polymorphism of the Regulator of G-protein signaling 2 gene (*RGS2*) in the mother has been implicated in preeclampsia as well as in the development of chronic hypertension after preeclampsia. The RGS2 protein acts as an inhibitor of physiological vasoconstrictive pathways, and a low RGS2 level is associated with hypertension and obesity, two conditions that predispose to preeclampsia. We genotyped the rs4606 polymorphism in 1339 preeclamptic patients and in 697 controls from the Finnish Genetics of Preeclampsia Consortium (FINNPEC) cohort to study the association of the variant with preeclampsia.

**Results:**

No association between rs4606 and preeclampsia was detected in the analysis including all women. However, the polymorphism was associated with preeclampsia in a subgroup of overweight women (body mass index ≥ 25 kg/m^2^, and < 30 kg/m^2^) (dominant model; odds ratio, 1.64; 95 % confidence interval, 1.10–2.42).

**Conclusions:**

Our results suggest that RGS2 might be involved in the pathogenesis of preeclampsia particularly in overweight women and contribute to their increased risk for hypertension and other types of cardiovascular disease later in life.

## Background

Preeclampsia is a complex syndrome of pregnancy characterized by hypertension, proteinuria and various metabolic disturbances resembling those seen in the metabolic syndrome [1]. It affects 2–8 % of pregnancies, and is one of the leading causes of maternal and perinatal mortality worldwide [2]. Insufficient placental perfusion is currently considered a central phenomenon in the development of preeclampsia [3]. However, multiple genetic [4, 5] and metabolic risk factors are likely implicated in the maternal response, which includes the development of systemic inflammation, endothelial dysfunction and an imbalance of angiogenic and antiangiogenic factors [3, 6–8]. The spectrum of preeclampsia symptoms and disease severity is wide and several pathogenic pathways are likely to contribute to different subtypes of the disease [9, 10]. Preeclampsia is associated with an increased risk of cardio- and cerebrovascular diseases [11]. Furthermore, being overweight predisposes to both cardiovascular diseases and preeclampsia [12], suggesting that these conditions may share genetic and other risk factors. Whether the genetic risk profile of women with preeclampsia differs according to the clinical heterogeneity of the syndrome remains undetermined.

**Fig. 1:**
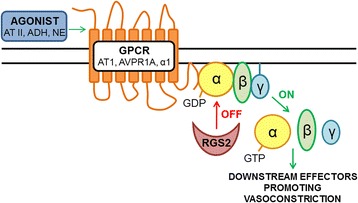
The role of the regulator of G-protein signaling 2 (RGS2) protein in vasoconstriction. Vasoconstrictive ligands, such as angiotensin II (AT II), vasopressin (ADH) and norepinephrine (NE), bind to their specific G-protein coupled receptors angiotensin II receptor type 1 (ATR1), vasopressin receptor 1A (AVPR1A) and α1-adrenergic receptor (α1) located in vascular smooth muscle cells. This leads to dissociation of the active subunits of the receptor and activation of the downstream effectors promoting vasoconstriction. The RGS2 protein enhances GTP hydrolysis in the Gα subunit inhibiting the dissociation of the subunits and therefore inhibiting vasoconstriction

One of the genes implicated in blood pressure regulation, the regulator of G-protein signaling 2 gene (*RGS2*), has recently been suggested to be associated with preeclampsia and with the development of chronic hypertension after pregnancy [13, 14]. Regulator of G-protein signaling (RGS) proteins control the activity of the Gα subunit located in the intracellular side of G protein-coupled receptors (GPCR). They enhance GTP hydrolysis in the Gα subunit and thereby inhibit the receptor (Fig. 1). RGS2 belongs to a group of RGS proteins that are involved in the regulation of blood pressure, and acts as an inhibitor of the GPCR-mediated vasoconstrictor signaling pathways [15] activated by vasoconstrictive ligands, such as angiotensin II, vasopressin and norepinephrine [16–18]. It is well known that there is increased sensitivity to angiotensin II in preeclampsia compared to normal pregnancy [19]. Also elevated levels of vasopressin and norepinephrine have been linked to preeclampsia [20–22]. The 3′ UTR C1114G polymorphism of *RGS2* (rs4606) is associated with low RGS2 levels [23] and has been connected to hypertension [23] and obesity [24]. In addition, rs4606 has been linked to anxiety disorders [25] and to posttraumatic stress disorder [26].

The aim of this study was to investigate whether rs4606 in *RGS2* is associated with preeclampsia in a Finnish case-control cohort, with specific focus on the potential impact of prepregnancy body mass index (BMI).

## Methods

### Subjects

We studied 1339 preeclamptic women and 697 women without preeclampsia from the Finnish Genetics of Preeclampsia Consortium (FINNPEC) cohort. The samples and data were collected during 2008–2011 at the five Finnish university hospitals. The inclusion criteria of the FINNPEC cohort were age above 18 years, a singleton pregnancy and sufficient language skills for understanding the research information and consent forms. In our study we excluded the women with a previous preeclamptic pregnancy or chronic or gestational hypertension, the pregnancies with small for gestational age infant and/or, placental insufficiency from the control group.

### Obstetric and perinatal data

The clinical data including information on preeclampsia in previous pregnancies, prepregnancy weight and height, smoking before and during pregnancy, blood pressure and proteinuria during pregnancy and perinatal outcomes were collected from the patient records. Prepregnancy weight was self-reported at the first antenatal visit, which usually takes place around 10th week of gestation and includes a measurement of current weight.

Preeclampsia was defined as systolic blood pressure ≥140 mmHg or diastolic blood pressure ≥90 mmHg measured at least twice after 20 weeks of gestation and proteinuria ≥0.3 g/24 h, or ≥1+ reading on dipstick in a random urine sample at least twice with no evidence of a urinary tract infection. Preeclampsia was defined superimposed if elevated blood pressure predated midpregnancy, including both women with chronic hypertension and *de novo* hypertension before midpregnancy. Preeclampsia was categorized severe in the presence of systolic blood pressure ≥160 mmHg, diastolic blood pressure ≥110 mmHg, proteinuria ≥5 g/24 h or clinically severe symptoms of preeclampsia, including clonus or respiratory distress. Each diagnosis was confirmed independently from medical records by a research nurse and a research physician. The study participants were categorized according to their prepregnancy BMI to normal weight (BMI <25 kg/m^2^), overweight (BMI ≥25 kg/m^2^ and <30 kg/m^2^) and obese (BMI ≥30 kg/m^2^). Placental insufficiency was defined as relative umbilical artery resistance index ≥ +2 SD or relative umbilical artery pulsatility index ≥ +2 SD for gestational age [27]. Relative birth weight (SD, birth weight standardized for sex and gestational age) was defined according to Finnish standards [28].

### Genotyping

A venous blood sample (36 mL) was drawn from all subjects. Genomic DNA was extracted from whole blood using the NucleoSpin Blood XL DNA extraction kit (Macherey-Nagel GmbH & Co.) or Chemagic Magnetic Separation Module I –machine (Chemagen) and subsequently stored at −20 °C. The genotyping was conducted at the Institute for Molecular Medicine Finland, Technology Centre, University of Helsinki, using the MassARRAY iPLEX method (Sequenom, San Diego, CA, USA).

### Statistical methods

Using the genetic power calculator [29] it was estimated that with a risk allele (G) frequency of 0.27 and preeclampsia prevalence of 5 %, our sample size of 1339 preeclampsia patients and 697 controls is sufficient to detect an effect size of 1.25 for the GC genotype and 1.5 for the GG genotype of rs4606 with power >80 % when α < 0.05 (dominant 1df test). Power calculation was based on the risk allele frequency of rs4606 in the European population according to the 1000 Genomes database [30], and on the effect sizes observed in the study by Kvehaugen et al. [13].

The Hardy-Weinberg test was performed using the PLINK [31] software. For the clinical characterization of the sample set, continuous variables were compared using the Mann–Whitney *U* test due to skewed distributions, and categorical variables using the chi-square test or the Fisher’s exact test. The allelic and genotypic association of rs4606 with preeclampsia was tested using binary logistic regression. Dominant, recessive and additive genetic models were utilized in the genotypic association test. For all tests, a p value <0.05 was considered statistically significant. Statistical analyses were performed with SPSS Statistics 22 software (IBM Corp.).

## Results

### Background characteristics of the study population

Basic maternal and perinatal background characteristics of the study participants are presented in Table 1. The preeclampsia group had a higher mean prepregnancy BMI and higher rates of gestational diabetes than the group of control subjects. Preeclamptic women also delivered on average earlier and had smaller placentas, and their infants had lower relative birth weights than those of the controls. There were significantly fewer women who smoked before pregnancy amongst the primiparous preeclamptic women compared to the primiparous controls.

**Table 1:** Maternal and perinatal background characteristics of the study groups

	Preeclampsia		Control			
	Primipara	Multipara	Primipara	Multipara	Primipara	Multipara
	(*n* = 987)	(*n* = 352)	(*n* = 377)	(*n* = 320)	*P* values	*P* values
Maternal characteristics						
Age, y	30 (26/33)	33 (30/38)	29 (25/32)	31 (28/35)	0.020	<0.001
Body mass index, kg/m^2^	23.6 (21.2/27.3) [985]	24.8 (22.1/29.4) [351]	23.0 (21.0/25.7)	22.8 (20.7/26.2)	0.006	<0.001
Highest systolic blood pressure, mmHg	165 (153/178)	166 (156/180)	126 (119/134)	124 (117/131)	<0.001	<0.001
Highest diastolic blood pressure, mmHg	109 (104/116)	109 (103/116)	83 (78/88)	81 (77/86)	<0.001	<0.001
Highest proteinuria, g/24 h	3.1 (1.4/6.2) [887]	2.9 (1.1/5.6) [305]	1.0 (0.8/1.6) [4]	0.6 [2]	N/A	N/A
Smoking before pregnancy	172 (18.3) [941]	48 (14.6) [328]	90 (24.8) [363]	44 (14.5) [304]	0.008	0.954
Smoking during pregnancy	58 (6.1) [948]	26 (7.8) [333]	28 (7.5) [371]	26 (8.4) [308]	0.345	0.769
Chronic hypertension	151 (15.3)	85 (24.1)	…	…	…	…
Gestational diabetes mellitus	116 (11.8)	69 (19.6)	29 (7.7)	24 (7.5)	0.030	<0.001
Pregestational diabetes mellitus	24 (2.4)	16 (4.5)	4 (1.1)	1 (0.3)	0.136	<0.001
Placental weight, g	495 (400/600) [951]	500 (390/615) [338]	590 (500/669) [363]	628 (554/720) [314]	<0.001	<0.001
Placental insufficiency	94 (9.5)	50 (14.2)	…	…	…	…
Perinatal characteristics						
Gestational age at birth, weeks	37.9 (35.4/39.3) [986]	37.4 (34.6/39.0)	40.6 (39.6/41.3)	40.3 (39.4/41.1)	<0.001	<0.001
Birth weight, g	2775 (2185/3280)	2790 (1882/3384)	3520 (3245/3851)	3730 (3416/4022)	<0.001	<0.001
Relative birth weight (SD)	−1.1 (−1.9/−0.4) [986]	−1.1 (−1.9/0.0) [350]	−0.2 (−0.7/0.5)	0.3 (−0.3/0.9)	<0.001	<0.001

Values for continuous variables are median (25th/75th percentile) and for categorical variables frequencies (%). Number of subjects with data is shown in brackets if different from the total number

### Association of the RGS2 rs4606 polymorphism with preeclampsia and body mass index

Rs4606 was successfully genotyped in 1324 (98.9 %) preeclamptic and in 694 (99.6 %) control women. The allele frequencies were found to be in Hardy-Weinberg equilibrium. The frequency of the risk allele G was 0.23. No association between rs4606 and preeclampsia was found under a dominant (Table 2), recessive or additive genetic model (data not shown).

**Table 2:** Association of the *RGS2* rs4606 genotypes with all preeclampsia patients and in groups divided by BMI

BMI category		n^a^	CC	GG or CG	OR (95 % CI)	*P*
All	Preeclampsia	1324	780 (58.9)	544 (41.1)	1.109 (0.919–1.338)	0.282
	Control	694	426 (61.4)	268 (38.6)		
Normal weight (BMI < 25 kg/m^2^)	Preeclampsia	784	484 (61.7)	300 (38.3)	0.971 (0.769–1.226)	0.806
	Control	480	293 (61.0)	187 (39.0)		
Overweight (BMI ≥ 25 kg/m^2^ and < 30 kg/m^2^)	Preeclampsia	310	162 (52.3)	148 (47.7)	1.635 (1.103–2.423)	0.014
	Control	159	102 (64.2)	57 (35.8)		
Obese (BMI ≥ 30 kg/m^2^)	Preeclampsia	227	133 (58.6)	94 (41.4)	0.913 (0.504–1.655)	0.764
	Control	55	31 (56.4)	24 (43.6)		

Binary logistic regression: preeclampsia patients vs control subjects, dominant model. Number of subjects and frequencies (%) are presented. OR indicates odds ratio, CI confidence interval and BMI body mass index. ^a^Altogether 1324 preeclamptic and 694 non-preeclamptic women were successfully genotyped. BMI information was missing for 3 preeclamptic cases (1 CC and 2 CG genotypes)

A significant association of the CG and GG genotypes with preeclampsia was seen in the subgroup of overweight women under a dominant model (Table 2). No association was detected when obese subjects were included in the analysis. The CG and GG genotypes were not statistically significantly associated with BMI categories in the control group.

In moderated multiple regression analysis where we investigated the effect of genotype (dominant model), categorical BMI and the interaction between the genotype and categorical BMI on the risk of preeclampsia, BMI as a categorical variable was found to significantly increase the risk (*p* < 0.001). Interaction analysis did not provide robust evidence on differing effects of the rs4606 risk genotype on preeclampsia risk in different BMI categories, although the p value for the interaction effect was close to significance (*p* = 0.069).

## Discussion

In this study, CG and GG genotypes of the *RGS2* 3′ UTR polymorphism rs4606 were not associated with preeclampsia when all study participants were included in the analysis. However, the association with preeclampsia was seen in the subgroup of overweight women.

The strengths of this study include a clinically well-characterized ethnically homogenous study population with extensive clinical and background information available on each study participant. The participants were recruited from all Finnish university hospitals and therefore could be considered representative of the Finnish population. Frequency of the risk allele G of rs4606 was lower in the Finnish sample set (0.23) than in the 1000 Genomes European data (0.27) [30] utilized in the power calculation, but our data set still had decent statistical power of 0.79 to detect effect sizes of 1.25 for the GC genotype and 1.5 for the GG genotype when α < 0.05 (dominant 1df test).

Although we detected association of CG or GG genotype with preeclampsia in overweight women, this association was not seen in obese women. The CG and GG genotypes were not associated with BMI categories in the control group, suggesting that the risk genotype increases preeclampsia susceptibility by a mechanism other than increasing BMI. Moderator analyses evaluating interaction between the rs4606 genotype and categorical BMI on preeclampsia risk were inconclusive. We speculate that obesity, a complex trait, which in itself is a risk factor for preeclampsia might override the influence of one genetic variant on preeclampsia susceptibility. Nonetheless, this finding needs further investigation in other clinically well-characterized preeclampsia cohorts.

Rs4606 has previously been associated with preeclampsia and recurrent preeclampsia in a Norwegian population [13]. The total sample size in the present study was somewhat smaller than that available in the study by Kvehaugen et al. [13], which may have contributed to our failure to replicate this finding. Another possibility is that the effects of rs4606 polymorphism on the risk of preeclampsia are modified by some population-specific genetic or other factors. Detailed clinical information was lacking in part of the Norwegian study population, and therefore their study could not assess the association of rs4606 genotypes with preeclampsia in overweight women. However, this subgroup of parturients should be examined also separately, since being overweight prior to pregnancy is a risk factor for preeclampsia [12, 32] and predicts later metabolic syndrome [33]. Furthermore, the changes in lipid and insulin metabolism seen in preeclampsia suggest a state of increased insulin resistance similar to the metabolic syndrome [1, 34] and persist several years postpartum [35, 36]. In accordance with these observations, prior preeclampsia elevates the risk of cardiovascular diseases and impaired glucose metabolism in later life [37–40]. Taking into account these apparent inter-linkages between obesity, insulin resistance and preeclampsia, it is possible that rs4606 is one of the genetic risk factors with small effect size that contribute to a maternal constitution susceptible to develop preeclampsia in the presence of a metabolic risk factor, overweight. Interestingly the −391 C to G substitution in the promoter of *RGS2* has been associated with metabolic syndrome in white European men [41] and the rs4606 CG or GG genotypes have been found to predict weight gain in young hypertensive men [24].

Several biological mechanisms, such as activation of renin-angiotensin system (RAS) and sympathetic nervous system, are involved in the development of obesity-related hypertension (reviewed in [42]). Adipose tissue is an important source for the components of the RAS system, which main effector is angiotensin II [43], a vasoconstrictor in the RGS2-inhibited pathway. Renal sympathetic system activation in obese individuals is marked by increased levels of another RGS2-inhibited vasoconstrictor, norepinephrine [44]. Taken together, being overweight increases the release of vasoconstrictive agents to which overweight women with low RGS2 levels might have impaired capacity to respond to.

The CG and GG genotypes of rs4606 have been linked to personality traits and brain function correlated with anxiety disorders [25] as well as to lower likelihood of benefiting from sertraline treatment to social anxiety disorder [45]. Overweight and obese pregnant women might constitute a subgroup that is especially vulnerable for comorbid anxiety [46], and anxiety and depression have been associated with the risk of preeclampsia [47, 48]. Unfortunately, we did not have any information on personality traits or anxiety disorders of the study subjects.

This study encourages further research exploring the role of *RGS2* in preeclampsia and its short- and long-term comorbidities such as obesity, cardiovascular disease and anxiety disorders. Heterogeneity of preeclampsia poses a challenge in candidate gene association studies. The identification of genetic variants that predispose to subtypes of preeclampsia demands large DNA collections, because the expected effect sizes of individual sequence variants on the preeclampsia risk are small [49]. To this end, large international collaborations with carefully characterized cohorts play a vital role.

## Conclusions

In this study rs4606, an *RGS2* 3′UTR polymorphism connected to low levels of RGS2, was not associated with preeclampsia. However, this polymorphism was associated with preeclampsia in a subgroup of overweight women. Our study suggests that the function of RGS2 could be one of the factors explaining the complex connection of preeclampsia and maternal overweight and warrants further investigation in other clinically well-characterized preeclampsia cohorts.

## Abbreviations

ADH: vasopressin
AT II: angiotensin II
ATR1: angiotensin II receptor type 1
AVPR1A: vasopressin receptor 1A
BMI: body mass index
FINNPEC: The Finnish Genetics of Preeclampsia Consortium
GPCR: G protein-coupled receptor
NE: norepinephrine
RAS: renin-angiotensin system
RGS: regulator of G-protein signaling
*RGS2*: regulator of protein signaling 2 gene
α1: α1-adrenergic receptor
